# Putative Bioactive Motif of Tritrpticin Revealed by an Antibody with Biological Receptor-Like Properties

**DOI:** 10.1371/journal.pone.0075582

**Published:** 2013-09-24

**Authors:** Raghava Sharma, Suvendu Lomash, Dinakar M. Salunke

**Affiliations:** 1 National Institute of Immunology, New Delhi, India; 2 Regional Centre for Biotechnology, Gurgaon, India; Indian Institute of Science, India

## Abstract

Antimicrobial peptides represent one of the most promising future strategies for combating infections and microbial drug resistance. Tritrpticin is a 13mer tryptophan-rich cationic antimicrobial peptide with a broad spectrum of activity whose application in antimicrobial therapy has been hampered by ambiguity about its biological target and consequently the molecular interactions necessary for its antimicrobial activity. The present study provides clues about the mechanism of action of tritripticin by using a unique monoclonal antibody (mAb) as a ‘physiological’ structural scaffold. A pool of mAbs were generated against tritrpticin and based on its high affinity and ability to bind tritrpticin analogs, mAb 6C6D7 was selected and characterized further. In a screening of phage displayed random peptides, this antibody was able to identify a novel antimicrobial peptide with low sequence homology to tritrpticin, suggesting that the mAb possessed the physico-chemical characteristics mimicking the natural receptor. Subsequently, thermodynamics and molecular modeling identified a core group of hydrophobic residues in tritrpticin arranged in a distorted’s’ shaped conformation as critical for antibody binding. Comparison of the mAb induced conformation with the micelle bound structure of tritrpticin reveals how a common motif may be able to interact with multiple classes of biomolecules thus extending the target range of this innate immune peptide. Based on the concurrence between thermodynamic and structural data our results reveal a template that can be used to design novel antimicrobial pharmacophores while simultaneously demonstrating at a more fundamental level the potential of mAbs to act as receptor surrogates.

## Introduction

It is now well established that antimicrobial peptides are an important component of the innate defenses in a wide variety of species [1-3]. They display broad spectrum of activity, rapid killing kinetics, and a lower incidence of generating resistance and are being increasingly explored as an alternative to conventional antibiotics especially as more and more drug resistant strains emerge [4]. As most of these peptides kill microorganisms rapidly as compared to other antibiotics, development of resistance to them also is less likely [5]. One of the large classes of antimicrobial peptides is comprised of cathelicidin family whose members have been shown to be active against many classes of pathogens [6]. First described in pigs, tritrpticin (VRRFPWWWPFLRR) is notable for its palindromic sequence, highly cationic nature and the central cluster of Trp residues [7]. It has been shown to be potent against a variety of microorganisms, fungi, and protozoa [8,9]. A number of structure-activity studies in the past, centered on its unusual sequence have been unable to reveal a consistent mechanism [10-14]. We earlier proposed that tritrpticin adopts a β-turn structure in aqueous buffer and undergoes functional activation through a conformational transition as an initial event in bacterial killing [10]. By contrast, Schibili et al. [11], reported that while tritrpticin shows a disordered structure in a Tris-based buffer, it adopts amphipathic turn structure in SDS micelles, so that its antimicrobial action might involve interactions with the cell membrane. In addition, results of an electrophysiological study suggested that tritrpticin has channel like activity in azolectin planar bilipids [12]. NMR and other spectroscopic techniques have used detergents to probe their interactions with tritrpticin and other related antimicrobial peptides and extend them to membrane lipids [15,16]. However, such studies presuppose bacterial membrane to be the sole target of these peptides, which is far from certain. Numerous reports on the closely related cathelicidin peptide indolicidin (ILPWKWPWWPWRR) have demonstrated its interaction with various molecules like DNA [17,18], topoisomerase I, calmodulin [19], and ATP [20]. Thus, it is conceivable that these peptides use their membrane binding property to enter the cytoplasm and exert their antimicrobial activity by attacking targets other than the membrane. In this context, the bioactive conformation of the peptide may exist independent of its structure in presence of membrane (mimetic micelles).

Lack of unique natural receptor has been the major hurdle not only in elucidating their mechanism of action but also in development of therapeutics based on such peptides. An alternative strategy in such cases has been the use of biomolecular scaffolds that can incorporate the properties of the receptor binding site. mAbs are prime candidates for such a role since their affinity and specificity can be naturally tailored and have indeed been used in varied scenarios to elucidate functionally relevant ligand-receptor interactions, constrain flexible molecules in their biologically active forms [21,22] and in few cases capture metabolites in their transition states [23]. Earlier studies from our laboratory demonstrated that an otherwise flexible peptide antigen could be held in a single conformation by three independent mAbs [24]. Further, we recently used a mAb as surrogate receptor to uncover a novel bioactive motif within the sequence of indolicidin [25].

This study describes an attempt to explore possible bioactive conformation(s) of tritrpticin by analyzing its three dimensional structure and interactions with a monoclonal antibody as a scaffold (non-membrane). The fact that this scaffold antibody was able to fish out a novel bactericidal sequence from a random peptide library underscores its ability to capture and represent interactions that confer antimicrobial properties on these peptides. The interactions of tritrpticin with mAb were therefore analyzed using a combination of different approaches involving thermodynamics and *in-silico* methods that correlated well to suggest structural/molecular determinants of tritripticin’s bioactivity.

## Materials and Methods

### Ethics Statement

All the experiments were performed after due approval from Institutional Animal Ethical Committee, National Institute of Immunology, New Delhi (IAEC#198/98) and following its guidelines.

### Peptide synthesis and conjugation to diphtheria toxoid (DT)

All the peptides were synthesized by solid-phase method on automated peptide synthesizer (431A; Applied Biosystems, Foster City, CA) using 9-fluorenylmethyloxycarbonyl chemistry on a *p-*hydroxymethylphenoxymethylpolysterene resin (Nova Biochem, San Diego, CA). Cleavage was performed using trifluoroacetic acid (Sigma-Aldrich, St. Louis, MO). Crude peptide was purified on a Delta, Pak C18 cloumn (Waters, Milford, MA) using a linear gradient of acetonitrile containing 0.1% trifluoroacetic acid. The identities of the peptides were characterized by mass spectrometry. Lysine was introduced at the N terminus of tritrpticin for its conjugation to diphtheria toxoid (DT), (Shantha Biotech, Hyderabad, India) using gluteraldehyde (Sigma-Aldrich). The gluteraldehyde solution in 20mM sodium acetate buffer, pH 5.5, was slowly added to the cold mixture of K-tritrpticin and DT with the molar ratio of 40:1 up to a final concentration of 0.1% in the reaction mixture. The reaction mixture was stirred for 10 h at 4°C. Synthesized conjugate was concentrated in a stirred cell (Millipore Corporation, Billerica, MA)

### Animals and immunization

Female BALB/c mice of 6-8 weeks of age were immunized with 200 µg of K-tritrpticin-DT conjugate emulsified with equal volume of complete Freund’s adjuvant (DIFCO Laboratories, Detroit, Michigan). The emulsified antigen was injected intraperitoneally in a volume of 200 µl per mouse. Animals were given two booster doses, each on day 28 after previous dose, and the sera samples were checked for the antibody titers after 15 days of the secondary booster. The highest responder mouse, selected for fusion, was given an intravenous injection of the antigen 3 days before the mouse was sacrificed for harvesting spleen cells for the generation of hybridomas.

### Generation and characterization of the mAbs

The sera from the immunized mouse were checked for anti-tritrpticin antibody titers. The highest responder of the group of mice was then sacrificed to harvest B cells from the spleen. The cells were allowed to fuse with the SP2/0 myeloma cells, maintained in log phase, in the presence of PEG1600 (Sigma-Aldrich). The cells were subjected to hypoxanthine-aminopterin-thymidine (Sigma) selection in DMEM (PAA Laboratories GmbH, Austria) in which only the hybrid cells are able to survive and grow. The supernatant of the wells with colonies of hybrid cells was screened for the presence of peptide recognizing Ab by ELISA, in which tritrpticin was used as coating antigen. The positive clones were further subcloned using limited dilution technique to ensure monoclonality.

### Sequencing of the variable regions of antibody heavy (V_H_) and light (V_L_) chains

A total of ~10^6^ to 10^7^ hybridoma cells were used for total mRNA extraction with TRIzol reagent (Invitrogen Life Technologies, Carlsbad, CA). cDNA of both the V_H_ and V_L_ chains of mAb 6C6D7 was synthesized from total RNA (~2 µg) using 10 U of reverse transcriptase (Promega, Madison, WI), 0.5 mM 3' primer (Mouse Ig primer set; Novagen, Madison, WI) and 0.2 mM dNTPS in total reaction volume of 50 µl. Single-stranded cDNA was amplified using the 5' primer (Mouse Ig primer set; Novagen), 1.5 mM MgCl_2,_ 0.2 mM dNTPs, and 1 U of *Taq*DNA polymerase (Promega) in total reaction volume of 50 µl. Bio-Rad Thermocycler (Berkeley, CA) was used for PCR with initial denaturation at 95°C for 3 min, followed by 30 cycles each at 94°C for 1 min, 58°C for 45 sec, 72°C for 1 min, and final incubation at 72°C for 7 min. A total of 3 µl of the PCR product was analyzed on 1% agarose gel. Subsequently, the PCR products were sequenced using their respective forward primers.

### Purification of the mAb from ascites

8 to 10 week old BALB/c mice were injected with incomplete Freund’s adjuvant (DIFCO Laboratories, Detroit, Michigan) after irradiation at 400 rad, 7 days before injecting hybridoma cells. Cells were washed and resuspended in phosphate buffered saline (PBS). A total of ~5 x 10^5^ to 5 x 10^6^ hybridoma cells were injected into each mouse. The ascites was tapped from the peritoneal cavity of the mice after 5-7 days. The Ab was purified in three-step purification protocol. The first step involved precipitation of the Ab by a 40% (v/v) ammonium sulfate cut. The precipitated Ab was resolubilized in 10 mM Tris, pH 8.5, and subjected to affinity chromatography on Protein-G sepharose column. The bound Ab was eluted with 50 mM Glycine–HCl buffer, pH 2.8, and subjected to anion exchange chromatography on DEAE column after concentrating. The bound protein was eluted with a gradient of NaCl, and purity of the Ab preparation was checked on SDS-PAGE. This preparation was used for all additional experiments. The concentration of the Ab was estimated by BCA kit (Thermo, Fisher Scientific, Waltham, MA) using BSA as the standard.

### Affinity measurements and thermodynamic analysis

Affinity measurements were based on surface plasmon resonance (SPR) technique, carried out on BIAcore 2000 (GE Healthcare). K-tritrpticin was amine coupled to a CM4 chip (~6 resonance units) and mAb 6C6D7 was injected as analyte with a 6 min association/15 min dissociation phase. Regeneration was achieved by 0.1 N HCl. Interaction curves were simultaneously fit using bivalent model in BIAevaluation program to determine K_D_ = k_diss_/k_ass_ and ΔG_eq_ = RTlnK_D_, R is the universal gas constant and T is the temperature in kelvin. The temperature sensitivity of Ag association and dissociation rates of the Ab was assessed on the basis of the Arrhenius plots. The slopes of the Arrhenius plots provide the activation energy (Ea) for the corresponding steps. The individual thermodynamic parameters were calculated using the following equations: ΔH_a/d_ = Ea-RT; ln(k_a/d_/T) = -ΔH_a/d_/RT + T∆S a_/d_/R + ln(K'/h). In these equations K' represents Boltzmann’s constant and h the Planck’s constant. Using these equations, the corresponding values of ΔH, T∆S, and ΔG for association and dissociation as well as the corresponding net values at equilibrium were calculated.

### Panning a Phage-displayed peptide library and assaying for antibacterial activity

A 12-mer peptide library (New England Biolabs) was panned with mAb 6C6D7 following the manufacture’s recommendations. Briefly, 0.1 mg of mAb 6C6D7 was coated in a 24-well plate, blocked with 0.5% BSA, and incubated with a phage library containing ~ 10^11^ virions. After washes with 0.1-0.7% tris buffered saline-tween, bound phages were eluted using 0.2 M glycine-HCl, pH 2.2, amplified and precipitated by 20% PEG 8000 containing 2.5 M NaCl. They were titrated and used as input for the next round. After four rounds of panning, individual viral plaques were randomly selected and sequenced for the 12-mer peptide.

Radial diffusion assay using double-layered agarose [26] involved incubating ~ 2 x 10^6^ mid-log phase bacterial cells with 50 nmol of peptide solution in nutrient-poor agarose at 37°C for 3 h. Overlaying with 3% tryptic soy broth agarose revealed circular bacteria-free zones. Minimum inhibitory concentration (MIC) was determined by broth microdilution method [27] in which ~10^4^ bacterial cells in 100 µl of 10 mM phosphate buffer were incubated for with serially diluted peptide solution in a 96-well microtiter plate for 2 hrs at 37°C. Addition of LB medium subsequently was used to ascertain the minimum peptide concentration sufficient to prevent bacterial growth, observable at OD_600_.

### ELISAs

The binding of the Ab from cell supernatant or in purified form, to the various peptide ligands was assayed by indirect ELISA. The peptide analogs were used as coating Ag at a concentration of 10 µg per well on 96-well immunosorbent plates. The wells were subsequently blocked with 5% lactogen to prevent nonspecific binding, and Ab was added at appropriate dilutions. HRP –labeled goat anti-mouse IgG was used as secondary Ab (Jackson Immuno Research) and o*-*phenylenediamine (Sigma) and H_2_O_2_ were used as peroxidase substrate. Absorbance was recorded at 490 nm after addition of 1 N H_2_SO_4_.

### Model building and refinement

The variable region of the mAb 6C6D7 was modeled from its sequence by using web based Rosetta Homology Modeling Server [28,29]. The structure obtained was energy minimized till convergence using the SANDER program of AMBER 9 [30]. The energy minimized structure was subjected to 8 ns completely unrestrained Molecular Dynamics (MD) simulation to analyze the stability of the model. Such a simulation done in the presence of solvent is a close approximation of the natural state wherein a flexible protein is expected to display conformational states in a dynamic equilibrium. Evaluation of the Cα RMSDs with reference to the initial energy minimized structure was considered to be a competent indicator of the degree of conformational diversity between the various states of the molecule generated during MD simulation. A stable RMSD trajectory with minimal fluctuations would indicate convergence of these states towards a stable structure, which can be considered close to the real scenario. A unique post-MD structure was arrived at by time averaging and energy minimizing the MD simulation from the point of stable trajectory through the end of the simulation. This structure was considered as the final stable Ab structure and used in *in-silico* docking simulations with tritrpticin.

### In silico simulations and analysis of peptide-antibody interactions

To predict the conformation and orientation of tritrpticin within the binding site of mAb 6C6D7, the program AutoDock4 [31,32] was used with default parameters. Starting conformation for tritrpticin was obtained from Protein Data Bank (PDB) code 1D6X. The Lamarckian genetic algorithm (LGA) of Autodock was used at the maximum number of allowable run cycles to search the conformational space for the best docking energy. The search for the docking site was restricted to the CDR regions. The largest allowable grid based on the united atom force field model was created to compute the potentials around the receptor to encompass the entire CDR region while simultaneously providing room for the ligand to rotate freely. The docked state and docking site itself were determined through an exhaustive search using the AutoDock genetic algorithm with 250 individual runs, each with 25 million energy evaluations and having a population size of 250. Preliminary screening and energy clustering of docked conformations was done using the ‘Analyze’ module of the program. The various docked conformations in the binding site were analyzed using the program PISA [33] in terms of binding free energy (ΔG_Total_), hydrophobic and hydrophilic components of ΔG, interface area, number of hydrogen bonds etc.

Molecular modeling and visualization was performed using PyMOL [34]. All the MD simulations were carried out with an explicit solvent with a 10 Å TIP3P water box and Na^+^ counterions beyond the boundary of the F_v_ or the F_v_-peptide structure using SANDER program of AMBER 9. Before all MD runs, the temperature was equilibrated to 300 K at constant volume conditions for 0.25 ns and another 0.25 ns of constant pressure conditions. The MD simulations were carried out with a time step of 2 fs and non-bonded interactions were cut off at 10 Å. A unique post-MD structure was arrived at by averaging and minimizing the trajectory conformations from region showing negligible RMSD fluctuations.

## Results and Discussion

### Generation and purification of anti-tritrpticin monoclonal antibody

Splenocytes from mice immunized with tritrpticin-diphtheria toxoid conjugate were used to raise anti-tritrpticin Ab secreting hybridomas as described in materials and methods. Although impressive polyclonal response was observed 2 weeks post first booster, a second booster was given to get mature high affinity antibodies and splenocytes were harvested 3 weeks afterwards. Post fusion, the hybridoma colonies could be observed in 5-7 days and by second week they were optimal for ELISA based detection of antibodies secreted in their culture supernatant (Figure S1A). Although the initial rounds of hybridoma screening resulted in 20-30% of the culture wells testing positive, they represented two unique clones 6C6 and 2A6. Both the clones demonstrated significant binding to tritrpticin, and negligible binding to BSA used as negative control (Figure S2).

**Figure 1:**
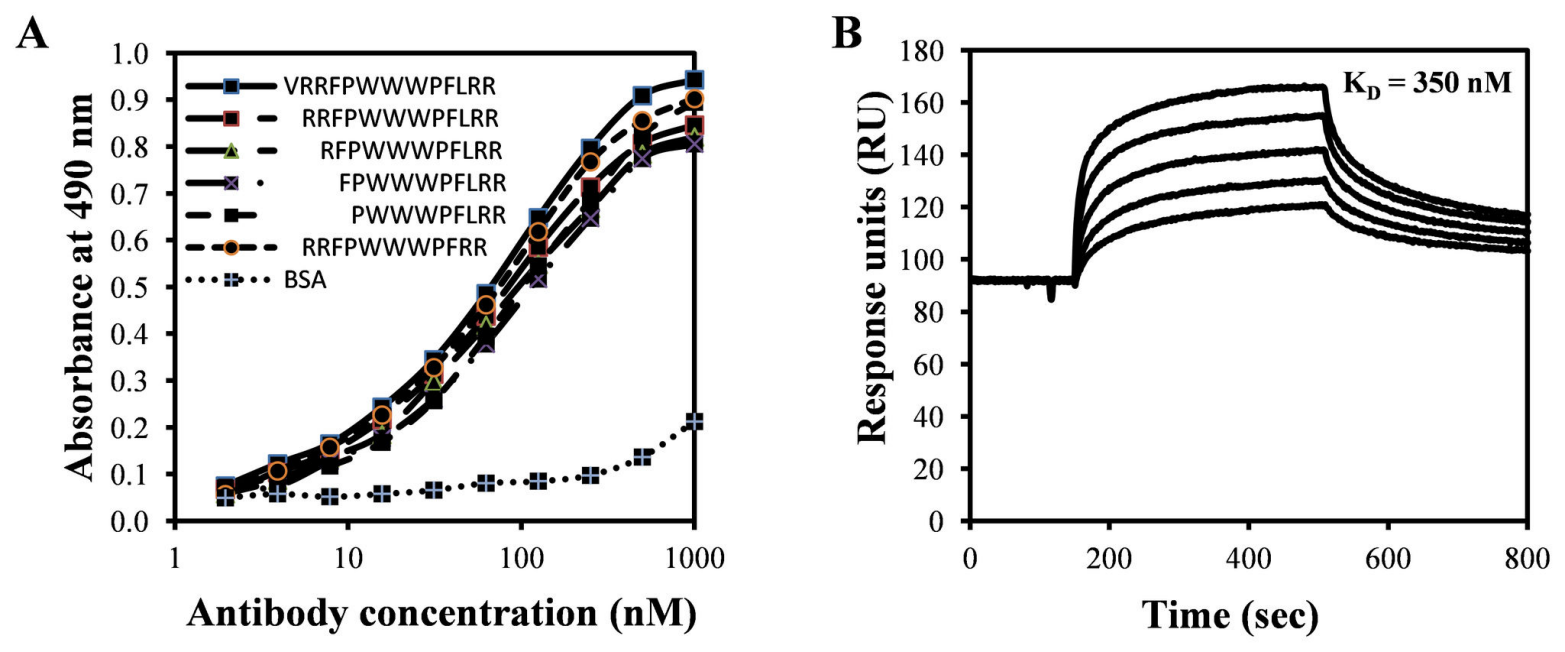
Binding analysis of 6C6D7. A. Direct binding ELISA demonstrating cross-reactivity of mAb 6C6D7 with N-terminal deletion analogs of tritrpticin. B. SPR sensorgram of binding of mAb (62.5-1000 nM) to K-tritrpticin on a CM-4 chip.

6 C6, subcloned as 6C6D7, showed higher levels of antibody secretion, and was used for further studies. Large quantities of mAb 6C6D7 were generated by inducing ascites in mice, and purified mAb was obtained through a combined ammonium sulfate fractionation, anion-exchange chromatography, and affinity chromatography procedure (Figures S1B and S1C). This preparation of Ab was used for all further experiments. In an indirect-binding ELISA where the peptides were directly immobilized on to the ELISA plate, mAb 6C6D7 could bind to tritrpticin and its N-terminal deleted antibacterial analogs at nanomolar concentrations (Figure 1A). This was confirmed and quantified by SPR based assay where the equilibrium dissociation constant (K_D_) for tritrpticin-mAb 6C6D7 binding was found to be 350 nM (Figure 1B).

We had earlier designed a perfectly symmetric tritrpticin variant by deleting Val-1 and Leu-11, which was significantly more active than parent molecule [14]. mAb 6C6D7 also showed binding to this peptide (Figure 1A), suggesting that it could recognize the determinants of antimicrobial activity of tritrpticin. Thus, we began characterizing the peptide-mAb interactions at a molecular level.

### Analysis of paratope of mAb 6C6D7

**Figure 2:**
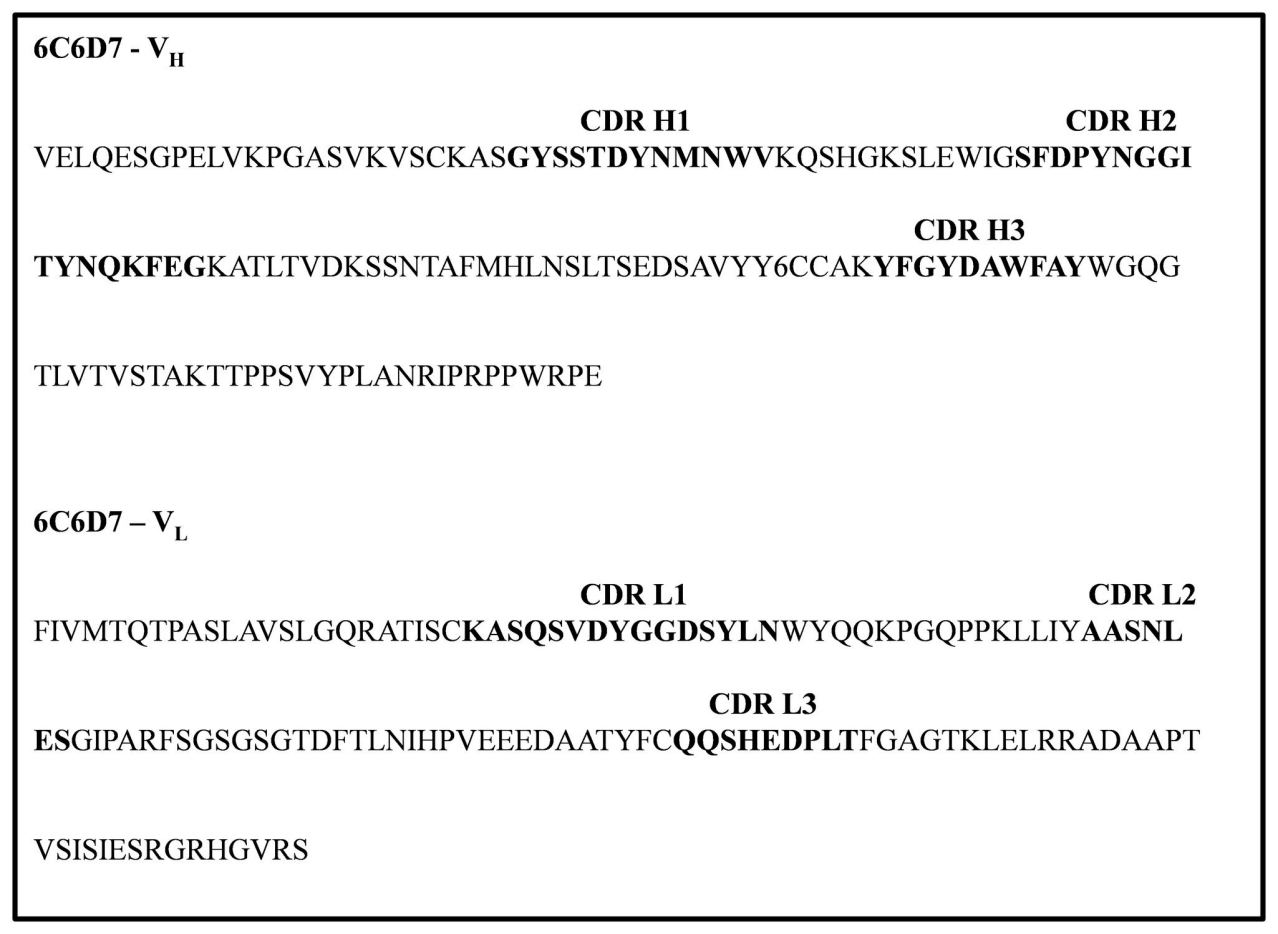
Sequencing of 6C6D7 light and heavy chain variable regions. Amino acid sequence of 6C6D7 light and heavy chain variable regions are shown with CDRs marked. The CDRs were determined from the gene sequence using IMGT database and IgG BLAST.

The variable regions of heavy (V_H_) and light (V_L_) chains of the anti-tritrpticin mAb 6C6D7 were sequenced by RT-PCR based protocol to determine the nature of residues that form the antigen binding site and also to allocate their germline origins. The International Immuno-genetics Database’s (IMGT) V-Quest tool [35] was used to identify the germ line origins of the light and heavy chains (Table 1). The L chain of mAb 6C6D7 belonged to the κ family which is predominant in mice and comprises of almost 95% of all mice L chains [36]. The V element of light and heavy chains showed 96.91% and 94.79% identity with their respective germline gene segments. While the J region of the light chain had 97.22% identity to its germline sequence, the H chain J region showed 97.92% identity with its germline origin. The CDR regions were determined by following the Chothia numbering scheme (Figure 2).

**Table 1:** Germline origin and CDRs of light and heavy chains of anti-tritrpticin mAb 6C6D7.

	**Heavy chain**	**Light chain**
Germline origin V	IGHV1S135*01	IGKV3-4*01
Germline origin J	IGHJ3*01	IGKJ5*01
CDR1	GYSSTDYNMNWV	KASQSVDYGGDSYLN
CDR2	SFDPYNGGITYNQKFEG	AASNLES
CDR3	YFGYDAWFAY	QQSHEDPLT

CDRs of heavy chain are rich in hydrophobic / aromatic amino acid residues.

The antibody hypervariable loops that contribute to the paratope were found to be rich in aromatic amino acid residues like tyrosine and phenylalanine especially in CDR H3. This compliments the peptide epitope that is similarly endowed with three tryptophan and two phenylalanine residues, thereby providing a surface for complex formation.

### Biopanning a phage displayed peptide library

**Figure 3:**
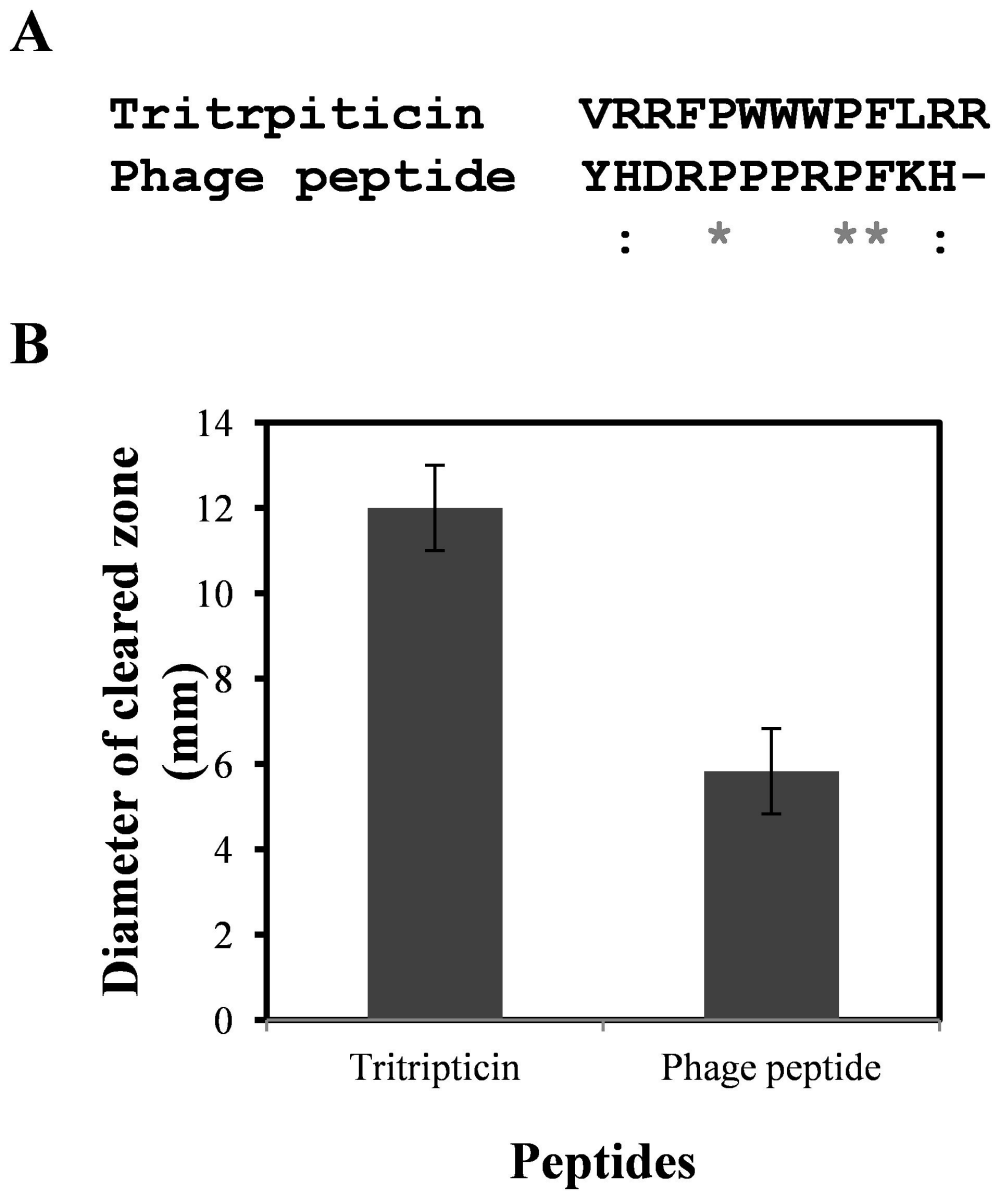
Novel antibiotic peptides picked by anti-tritrpticin mAb 6C6D7. A. Alignment of tritrpticin with novel sequence panned from random peptide library by mAb 6C6D7. B. Comparison of antibiotic activities of tritrpticin and phage displayed peptide against *E. coli* BL21λD3 in a radial diffusion assay. An unrelated peptide EHGTPPRVMSSM was used as negative control and as expected did not led to the formation of clearance zone (not shown in the figure). 50 nmol of peptides were used in the assay.

Phage display describes a selection technique in which a library of random peptides is expressed on a phage virion, by incorporating the genetic material encoding each variant in a DNA cassette that is fused to the gene encoding outer coat protein of an M13 phage. This allows rapid partitioning based on binding affinity to a given target molecule by an *in vitro* selection process called panning. We tested our hypothesis that the nature of the antigen combining site should be reflected in the peptide(s) selected by biopanning of a random peptide library. A 12mer peptide library was chosen for screening as it is approximately the same length as tritrpticin. The biopanning protocol followed is detailed in materials and methods. After the first round, bound phages were pre-eluted at pH 2.8 and then at pH 2.2, thus ensuring that the eluted phages have higher binding affinities for the antibody. After 4 rounds a sequence named 12PhD1 was identified. Despite sharing less than 30% homology with tritrpticin at the level of primary structure (Figure 3A), it turned out to have broad spectrum antibiotic activity (Figure 3B, Table 2 and Figure S3). Careful examination of sequence of phage peptide revealed the presence of Pro-Arg-Pro motif. Interestingly this motif is also present in drosocin and apidaecin, antimicrobial peptides from drosophila and honey bee respectively where the Pro-Arg-Pro motif is believed to be critical for activity [37]. Interestingly, different families of poly proline recognition domains (PRD) like SH3 (Src-homology 3) domains, WW (named after two highly conserved tryptophan residues) domains, EVH1 (enabled vasodilator-stimulated-protein homology) domains, GYF (characteristic gly-tyr-phe triad) domains are known to interact with proline rich core motifs through hydrophobic interactions between key proline residues of the ligand and highly conserved hydrophobic pockets in the domain [38-43]. Given the preponderance of apolar residues in both the tritrpticin’s epitope and the antibody paratope, it appeared plausible that the interactions of the mAb with both the peptides may be hydrophobic in nature. Within the limited repertoire of phage display library, the fact that mAb 6C6D7 could fish out a novel peptide that had lower though qualitatively similar broad spectrum antimicrobial activity as tritrpticin indicated that it was able to capture the essence of their microbicidal property. Next, we proceeded to characterize the molecular interactions that may provide a clue to the antimicrobial activity of tritrpticin using a combination of thermodynamics and molecular dynamics.

**Table 2:** MIC values of tritrpticin and phage peptide against different bacterial strains.

**Bacterial strain**	**Tritrpticin (μM**)	**Phage peptide (μM**)
*E. coli* BL21λD3	3.12	400
*E. coli* ATCC25922	3.12	400

### Kinetics and thermodynamics of peptide-antibody binding

The binding kinetics and thermodynamics of this receptor-ligand system was analyzed through SPR based assay. A typical sensorgram at 25°C is shown in Figure 1B. The binding experiment was carried out at different temperatures, ranging from 15°C to 35°C and equilibrium binding constants were calculated at each temperature (Table 3). At 25°C, mAb 6C6D7 binds to the peptide antigen with a k_ass_ of 3.91 x 10^4^ M^-1^s^-1^and k_diss_ of 13.7 x 10^-3^ s^-1^ resulting in the affinity of 350 nM. There is a minor effect of temperature on binding; while there is only 2 fold increase in the association rate constant, there is approximately 7 fold increase in k_diss_ that results in 3 fold decrease in affinity (K_D_) on increase in temperature from 15°C to 35°C.

**Table 3:** Affinity of mAb 6C6D7 to K-tritrpticin at different temperatures obtained by SPR based binding assays.

**Temperature (°C**)	**k_a_ x 10^4^ (M^-1^s^-1^**)	**k_d_ x 10^-3^ (s^-1^**)	**K_D_ x 10^-7^ (M**)	**ΔG_eq_ (kcal/mol**)
15	2.44	5.15	2.11	-8.80
20	3.79	11.5	3.03	-8.74
25	3.91	13.7	3.50	-8.80
30	4.42	23.1	5.45	-8.68
35	5.41	34.3	6.34	-8.73

k_a_ – Rate of association, k_d_ – Rate of dissociation, K_D_ – Dissociation constant, ΔG_eq_ – Gibbs free energy at equilibrium.

**Figure 4:**
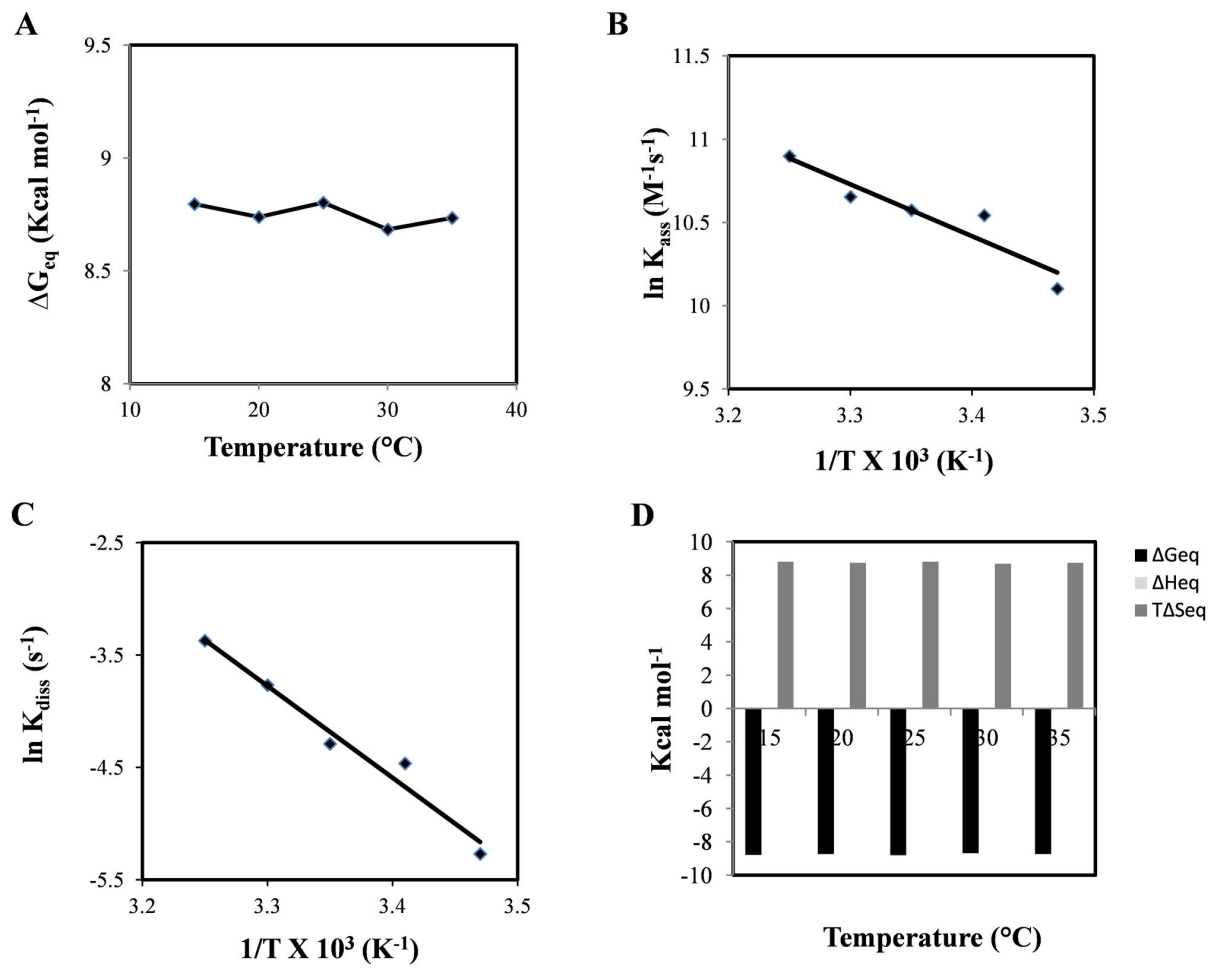
SPR based thermodynamics of antigen – antibody interaction. A. Changes in Gibbs free energy of binding (∆G) at different temperatures (15°C - 35°C). B. Arrhenius plot for association. C. Arrhenius plot for dissociation. D. Individual contributions of enthalpy (∆H) and entropy (T∆S) to equilibrium free energy of binding (∆G), at temperatures ranging from 15°C to 35°C. Bars corresponding to ∆H are not visible in the figure because of their negligible contribution.

Thermodynamic basis of peptide antibody interaction was studied by calculating different energetic parameters including change in Gibbs free energy (ΔG), enthalpy (ΔH) and entropy (T∆S) during association and dissociation phase and at equilibrium. The relative contributions of these components indicate the possible modes of binding. The change in Gibbs free energy of equilibrium upon binding of tritrpticin, as a function of temperature is shown in Figure 4A and was ~ -9 kcal/mol, which is typical range for binding of small ligands to proteins [44]. As can be seen in Figure 4A and Table 3 the binding of 6C6D7 to tritrpticin is characterized by negligible change in ΔG_eq_ with respect to temperature that suggests that the antibody does not undergo any major conformational alteration during the binding of peptide antigen. The antigen binding site appears to lack conformational flexibility, indicating that mAb was antigen educated; in the sense that paratope was restricted to only those conformations that possessed improved antigen complementarity.

To further examine the temperature sensitivity of peptide binding, enthalpy and entropy contributions towards free energy for association and dissociation phase as well as equilibrium were calculated through the Arrhenius plot. As is evident from these plots, the dissociation phase of antibody peptide binding appears to be more sensitive to variation in temperature in comparison with the association phase (Figure 4B and 4C). It is interesting to note that, at all the temperatures entropy is highly favorable (Figure 4D) more than compensating for marginally unfavorable enthalpy, suggesting that the binding is primarily driven by hydrophobic interactions involving burial of nonpolar groups. This further strengthens our view that mAb 6C6D7 is well adapted to accommodate antimicrobial motif, without the need for any significant conformational readjustment. Such an inference is also consistent with prior observations. For example, the crystal structure of 1E9, an antibody raised against an analog of the transition state in Diels-Alder cycloadditions, reveals a binding pocket preorganized to maximize shape complementarity with the hapten through a combination of van der Waals contacts, π-stacking, and electrostatic interactions [45]. Here also, the thermodynamics revealed that binding is entropically driven with unfavorable enthalpy contribution. Also, the lack of variation in kinetics of binding as a function of temperature indicates the absence of flexibility in the antigen combining site.

### *In silico* structural studies of peptide-antibody interaction

**Figure 5:**
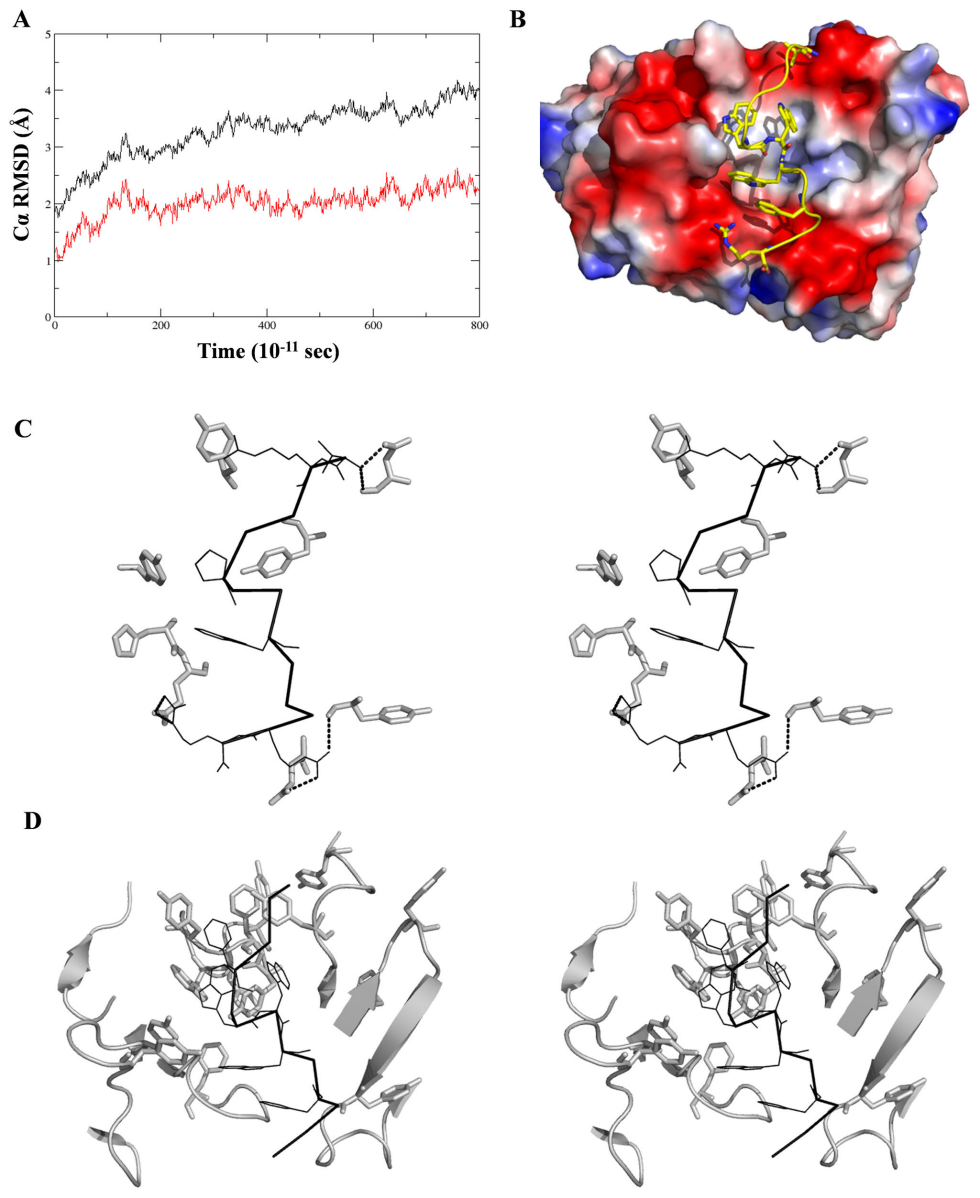
*In- silico* binding analysis of tritrpticin to mAb 6C6D7. A. RMSDs of Cα atoms of the CDRs and tritrpticin (black) and that of whole 6C6D7 Fv and tritrpticin (red) during the 8 ns MD simulation. The averaged structure from last 6 ns run was energy minimized and used for further analysis. B. Surface view of peptide – mAb interaction, shaded according to electrostatic potential (blue to red indicating positive to negative, respectively) showing complementarity of shape and charge. Also can be seen the three tryptophans buried in the hydrophobic cavity shielded from solvent. C. Stereoscopic representation of binding pocket containing bound peptide. Interacting residues for peptide are shown as ribbon and for Fv as sticks. The polar interactions obtained by PISA are shown as dotted lines. D. Stereo view of the binding pocket showing preponderance of aromatic residues both in paratope (ribbon) and epitope (sticks).

In an attempt to correlate the thermodynamics and energetics of the recognition of anti-bacterial motif with the nature and composition of antigen combining site and to gain insight in to the conformational properties of the antimicrobial motif in the context of mAb 6C6D7 *in-silico* methods were used. The mAb structure was modeled from its sequence using the Rosetta Antibody Homology modeler and the structure optimized as described in the materials and methods. The final model was structurally stable (Figure S4) and was used for elucidating the probable conformation(s) adopted by antibody bound tritrpticin using the program AutoDock4. Within the constraints on torsional degrees of freedom, the peptide was kept as flexible as possible, whereas the Fv model had to be held rigid. The lowest energy docked structure of tritrpticin bound 6C6D7 was energy minimized and subjected to 8 ns MD simulation to analyze its structural stability. The Cα RMSDs of the CDRs and the peptide together as well as that of the whole system, revealed that in both the cases, the trajectory stabilizes after initial 2 ns demonstrating negligible fluctuations for the rest of the 6 ns (Figure 5A). The ensemble of conformations for the last 6 ns was averaged, energy minimized and used for further analysis.

Analysis of docked simulation in terms of free energy changes (ΔG_Total_) yielded a value of -9.16 kcal/mol for tritrpticin-Fv 6C6D7 complex formation. This correlated quite well with the thermodynamic analysis presented above (-8.8 kcal/mol) validating the tritrpticin bound mAb structure (Table 4). Additional support to the docked structure came from the significantly greater ΔG_Hydrophobic_ contributions (~ 70% of ΔG_Total_) over ΔG_H-bonds_ that re-emphasized the predominant non-polar nature of the mAb-peptide complex. Role of individual residues of tritrpticin that interacted with mAb 6C6D7 was observed by analyzing the percent buried surface area of each residue. The three central tryptophans along with Phe 4 play significant role in binding the peptide. Trp-6 in particular is almost completely solvent shielded (Figure 5B). Tritrpticin buried itself into the hydrophobic cavity of the antibody giving the buried surface area of ~ 740 Å^2^, and adopted a distorted’S’ shaped conformation with three half-turns each at Pro-5, Trp-7 and Leu-11 (Figure 5B). N-terminal Val and both of the C-terminus Arg contributed towards binding by forming hydrogen bonds (Figure 5C). Significant contribution towards complex formation also came from placement of three Trp residues, Trp-6 in particular was surrounded by Tyr residues from the CDRs of the H chain (Figure 5D). It has been reported that it is not unusual for the antibodies to interface a larger antigen but concentrate binding interactions towards few residues that in some cases may be important for physiological function of antigen. An example is the high affinity binding of antibody V2D2 to indolicidin (25). Even though it is a 13mer peptide, the five Trp residues together account for almost all of the peptide antibody interactions. Yet another example is the binding of antibody 447-52D to the hypervariable V3 loop of the HIV gp 120. Here also the antigen is a 12mer peptide, but the major contribution to binding comes from ‘GPXR’ motif that is required for the interaction of the viral protein with the host receptor CCR5 [46]. Similar results have been obtained from analyzing the binding of human growth hormone with its natural receptor by mutational analysis [47]. Only a small set of hydrophobic contacts that formed complementary surfaces were found to be responsible for about three quarters of binding energy.

**Table 4:** Comparison of Gibbs free energy of binding of tritrpticin to mAb 6C6D7 as obtained by computational and SPR based studies and percent buried surface area of each residue of tritrpticin during interaction with mAb 6C6D7as calculated by PISA.

**Comparison of Free energy of binding**	
ΔG_SPR_	-8.8 kcal/mol
ΔG_*in silico*_	-9.16 kcal/mol
**% buried surface area of each residue**	
Val-1	50.65
Arg-2	0.97
Arg-3	0
Phe-4	56.41
Pro-5	42.22
Trp-6	90.37
Trp-7	62.41
Trp-8	54.88
Pro-9	0
Phe-10	11.66
Leu-11	22.11
Arg-12	59.73
Arg-13	37.47

Structural analysis of antigen binding pocket showed clustering of aromatic residues (Figure 5D) suggesting that the recognition of anti-bacterial motif is because of the hydrophobic interactions. Detailed analysis of binding site and interactions revealed the arrangement of multiple aromatic residues forming a hydrophobic cavity lined with negative charges at the brim, this unique organization of hydrophilic and hydrophobic patches mimics not only membrane like environment but also other possible intracellular target(s); like DNA in case of buforin II [48]. Similar structural features have been observed in few other cases. For example, the crystal structure of C2 domain of factor VIII complexed with its neutralizing antibody reveals that the antibody interacts with the hydrophobic residues that form the membrane association surface of factor VIII, and is thus capable of mimicking a membrane interface like environment [49].

## Conclusions

Using mAb as a structural scaffold, we have elucidated a structural motif in tritrpticin that is associated with its antimicrobial activity. Both the thermodynamic and *in-silico* binding data concur remarkably in terms of binding free energy to suggest that the peptide antibody interactions are hydrophobically driven, primarily by aromatic residues abundant in both epitope and paratope. While it may not be clear whether membrane is the actual target for tritrpticin, structural studies with mAb 6C6D7 indicate that it may be able to mimic a physiological binding environment independent of the biological class of the target receptor macromolecule(s). Thus, it seems plausible that the same cluster of hydrophobic residues at the core of tritrpticin sequence may mediate its interactions with membrane lipids, proteins and nucleic acids and therefore form the structural backbone of its biological activity. This is similar to the action of the related peptide indolicidin which has been shown to act on bacterial membranes as well as intracellular biomolecules like DNA, ATP and calmodulin [17-20]. The role of hydrophobic residues, especially tryptophan may be important in providing stacking interactions with the nitrogen bases of DNA, partitioning in lipids and/or interacting with enzymes with hydrophobic active sites that exclude water to provide a snug fit to their substrates. It is interesting to note that the peptide selected by mAb 6C6D7 from the phage-displayed library contained a Pro-Arg-Pro motif that is also associated with antimicrobial activity. Incidentally, Pro-Arg rich antimicrobial peptides are also known to cross the bacterial membrane and act intracellularly [50]. It is therefore, tempting to speculate that a common structural motif and/or set of interactions, as captured by mAb 6C6D7, underlie the activity of two classes of antimicrobial peptides, which may provide leads to design of novel antimicrobial compounds. More importantly our results demonstrates the utility of mAbs to effectively mimic receptors from varied biomolecular classes and identify underlying motifs associated with ligand activity.

## Supporting Information

Figure S1 **Generation and purification of mAb 6C6D7.** A. ELISA profile of initial screening of anti-tritrpticin monoclonal antibodies. Clone 6C6 was selected for further experiments due to its ability to secrete high levels of antibody. B. Protein G sepharose elution profile. C. SDS-PAGE profile of purification. LMW refers to Low molecular weight marker.
(TIF)

Figure S2 **ELISA based binding of clone 6C6 (A) and 2A6 (B) to tritripticin.** Both the clones were not able to bind indolicidin and BSA (used as negative control).
(TIF)

Figure S3 **Comparison of antimicrobial activity of tritripticin and phage displayed random peptide picked by mAb 6C6D7 against different bacterial strains.** An unrelated peptide EHGTPPRVMSSM was also used as negative control and as expected did not led to the formation of clearance zone (not shown in the figure). 50 nmoles of peptides were used for assay.
(TIF)

Figure S4 **RMSDs of Cα atoms of the CDRs (black) and that of whole 6C6D7 Fv (red) during the 8 ns MD simulation.** The averaged structure from last 4 ns run was energy minimized and used for docking studies.
(TIF)
