# Supplementary figures and images for: Putative Bioactive Motif of Tritrpticin Revealed by an Antibody with Biological Receptor-Like Properties
Source: PLoS One. 2013 Sep 24;8(9):e75582. doi: 10.1371/journal.pone.0075582 (PMC3782441; doi:10.1371/journal.pone.0075582)

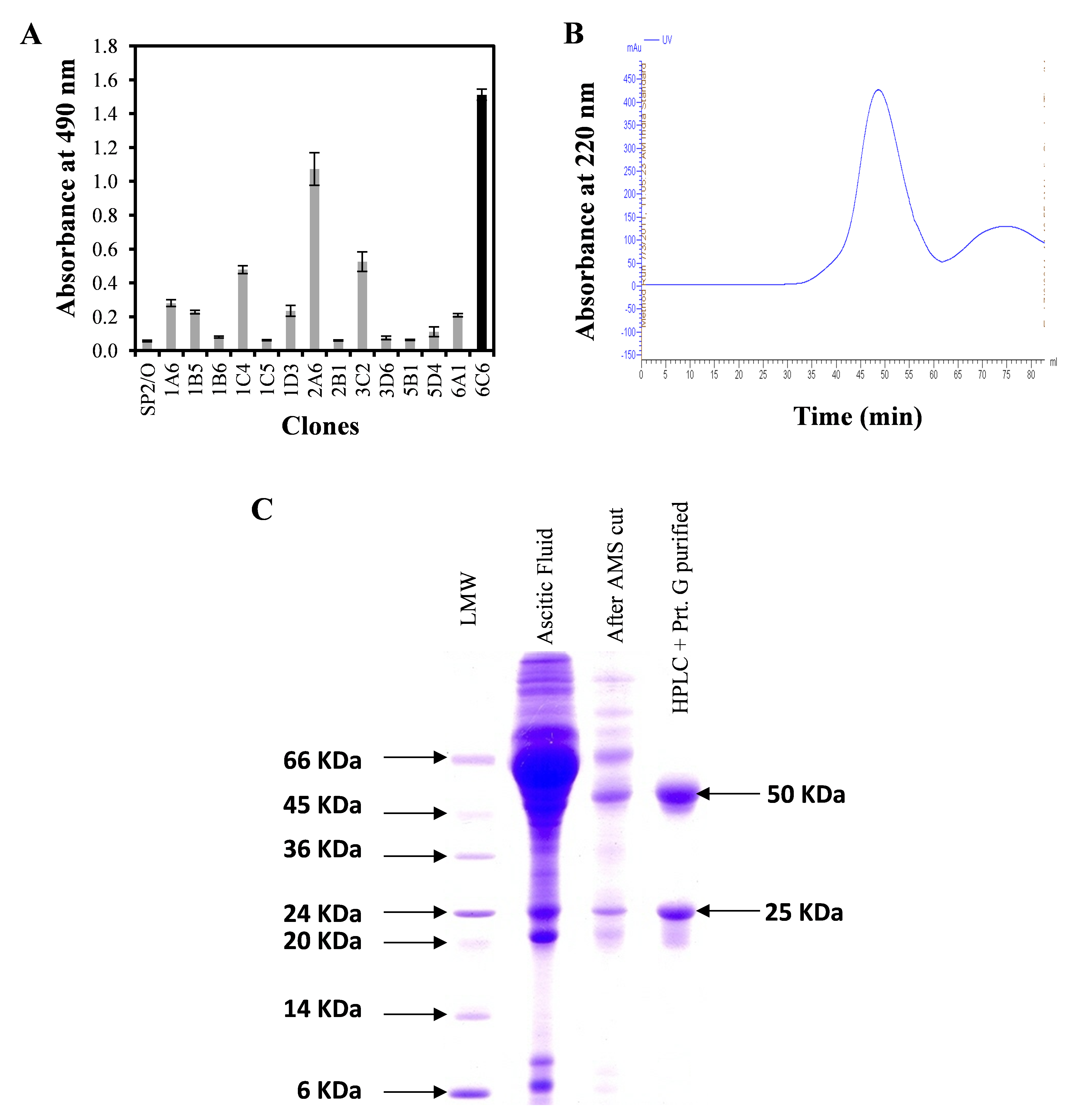

Supplement: Figure S1 — Generation and purification of mAb 6C6D7. A. ELISA profile of initial screening of anti-tritrpticin monoclonal antibodies. Clone 6C6 was selected for further experiments due to its ability to secrete high levels of antibody. B. Protein G sepharose elution profile. C. SDS-PAGE profile of purification. LMW refers to Low molecular weight marker. (TIF) [file pone.0075582.s001.tif]

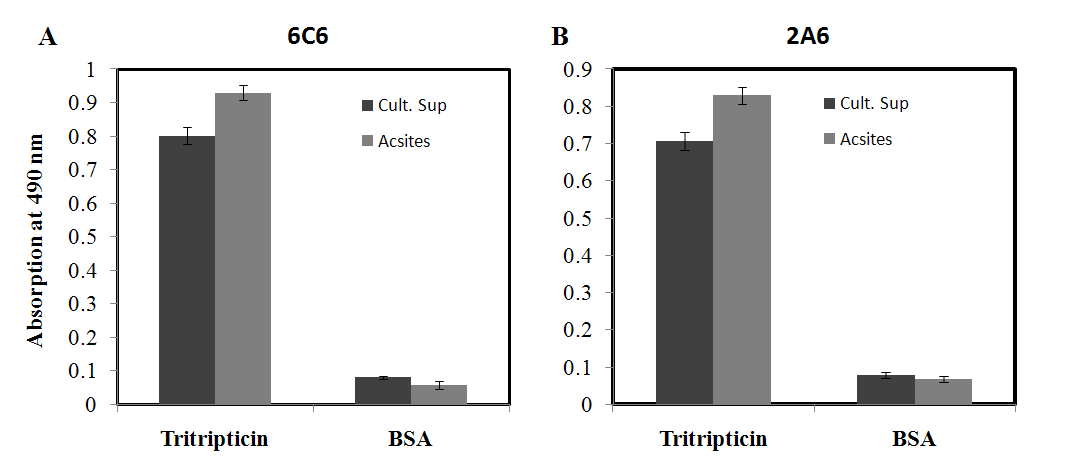

Supplement: Figure S2 — ELISA based binding of clone 6C6 (A) and 2A6 (B) to tritripticin. Both the clones were not able to bind indolicidin and BSA (used as negative control). (TIF) [file pone.0075582.s002.tif]

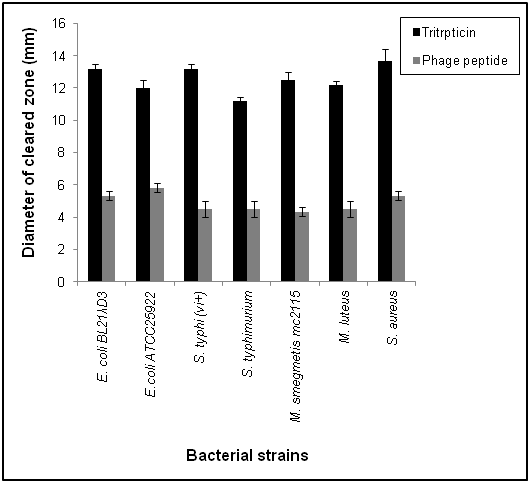

Supplement: Figure S3 — Comparison of antimicrobial activity of tritripticin and phage displayed random peptide picked by mAb 6C6D7 against different bacterial strains. An unrelated peptide EHGTPPRVMSSM was also used as negative control and as expected did not led to the formation of clearance zone (not shown in the figure). 50 nmoles of peptides were used for assay. (TIF) [file pone.0075582.s003.tif]

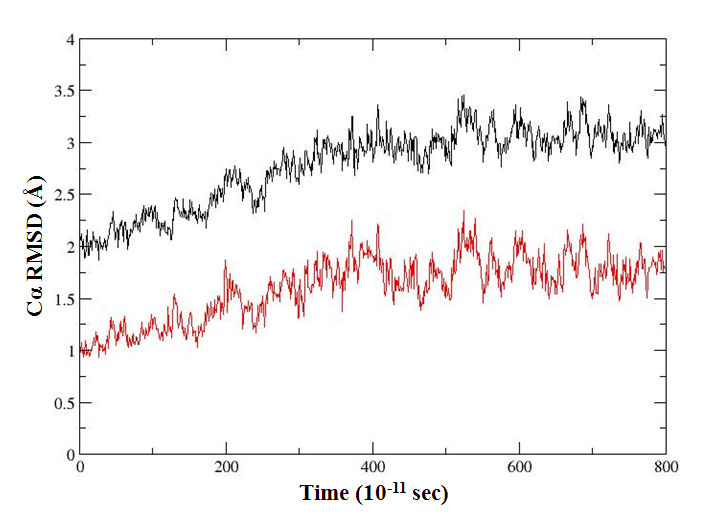

Supplement: Figure S4 — RMSDs of Cα atoms of the CDRs (black) and that of whole 6C6D7 Fv (red) during the 8 ns MD simulation. The averaged structure from last 4 ns run was energy minimized and used for docking studies. (TIF) [file pone.0075582.s004.tif]
